# Supplementary material for: Validation and measurement invariance of the Chinese version of the academic self-efficacy scale for university students
Source: PeerJ. 2024 Sep 24;12:e17798. doi: 10.7717/peerj.17798 (PMC11432435; doi:10.7717/peerj.17798)
Supplement: Supplemental Information 2 [file peerj-12-17798-s002.pdf]

## Academic Self-efficacy Scale

The following are the kinds of statements that people use to describe themselves. Read each one carefully and decide the extent to which each statement applies to you. There are no right or wrong answers. For each statement encircle the number which best describes you (**1-strongly agree** – **7-strongly disagree**). Please respond to all items.

1. I am confident that I can achieve good exam results if I really put my mind to it.*
2. If I don’t understand an academic problem, I persevere until I do.*
3. When I hear of others who have failed their exams, this makes me all the more determined to succeed.*
4. I am confident that I will be adequately prepared for the exams by the time they come around.*
5. I tend to put off trying to master difficult academic problems whenever they arise.
6. No matter how hard I try, I can’t seem to come to terms with many of the issues in my academic curriculum.
7. I am convinced that I will eventually master those items on my academic course which I do not currently understand.*
8. I expect to give a good account of myself in my end-of-semester exams.*
9. I fear that I may do poorly in my end-of-semester exams.
10. I have no serious doubts about my own ability to perform successfully in my exams.*
