# Supplementary material for: Validation and measurement invariance of the Chinese version of the academic self-efficacy scale for university students
Source: PeerJ. 2024 Sep 24;12:e17798. doi: 10.7717/peerj.17798 (PMC11432435; doi:10.7717/peerj.17798)
Supplement: Supplemental Information 3 [file peerj-12-17798-s003.pdf]

Academic Self-efficacy

学习自我效能问卷

1. 我有信心，如果我用心去学习，我可以取得好的考试成绩。*
2. 如果我不理解一个学习上的问题，我会一直钻研，直到理解为止。*
3. 当我听说别人考试失败时，我更加坚定了要成功的决心。*
4. 我相信，到了考试的时候，我将为考试做好了充分准备。*
5. 每当学习中出现了困难的问题时，我往往会将它们先放旁边，推迟尝试去理解与掌握它们。
6. 无论我如何努力，我似乎都无法理解学习中的许多问题。
7. 我相信，我最终会掌握学习中那些我目前不理解的问题。*
8. 我希望能在学期末的考试中给自己一个好的交代。*
9. 我担心期末考试可能会考砸。
10. 我对自己在考试中取得好成绩的能力没有太大的怀疑。*

1.非常同意
2. 同意
3. 比较同意
4. 中性
5. 比较不同意
6. 不同意
7. 非常不同意

评分：

一些条目（*）反向计分后，最后得分范围 10~70 分，分数越高说明学习自我效能越强。
